# Dosing of Electrical Parameters in Deep Brain Stimulation (DBS) for Intractable Depression: A Review of Clinical Studies

**DOI:** 10.3389/fpsyt.2018.00302

**Published:** 2018-07-11

**Authors:** Rajamannar Ramasubbu, Stefan Lang, Zelma H. T. Kiss

**Affiliations:** Department of Psychiatry and Clinical Neurosciences, Cumming School of Medicine, Hotchkiss Brain Institute, University of Calgary, Calgary, AB, Canada

**Keywords:** electrical stimulation, deep brain stimulation, treatment resistant depression, stimulation parameters, stimulation dosimetry

## Abstract

**Background:** The electrical parameters used for deep brain stimulation (DBS) in movement disorders have been relatively well studied, however for the newer indications of DBS for psychiatric indications these are less clear. Based on the movement disorder literature, use of the correct stimulation parameters should be crucial for clinical outcomes. This review examines the stimulation parameters used in DBS studies for treatment resistant depression (TRD) and their relevance to clinical outcome and brain targets.

**Methods:** We examined the published studies on DBS for TRD archived in major databases. Data on stimulus parameters (frequency, pulse width, amplitude), stimulation mode, brain target, efficacy, safety, and duration of follow up were extracted from 29 observational studies including case reports of patients with treatment resistant unipolar, bipolar, and co-morbid depression.

**Results:** The algorithms commonly used to optimize efficacy were increasing amplitude followed by changing the electric contacts or increasing pulse width. High frequency stimulation (>100 Hz) was applied in most cases across brain targets. Keeping the high frequency stimulation constant, three different combinations of parameters were mainly used: (i) short pulse width (60–90 us) and low amplitude (0–4 V), (ii) short pulse width and high amplitude (5–10 V), (iii) long pulse width (120–450 us) and low amplitude. There were individual variations in clinical response to electrical dosing and also in the time of clinical recovery. There was no significant difference in mean stimulation parameters between responders and non-responders suggesting a role for stimulation unrelated factors in response.

**Conclusions:** Although limited by open trials and small sample size, three optimal stimulation parameter combinations emerged from this review. Studies are needed to assess the comparative efficacy and safety of these combinations, such as a registry of data from patients undergoing DBS for TRD with individual data on stimulation parameters.

## Introduction

Deep brain stimulation (DBS) is an emerging investigational treatment for patients with treatment resistant depression (TRD). Based on evidence from open trials, DBS is an effective, safe, and reversible treatment for TRD. To date, there are several published reports of patients with TRD who improved with DBS applied to various brain targets (1–30). While these reports offer promise to millions of patients with TRD (i.e., 10–20% of patients with depression), there remain several unanswered questions. Although open label 50% response rates at 6 month time periods are promising, one sham control trial involving ventral capsule/ventral striatum (VC/VS) did not demonstrate benefit in comparison to sham stimulation at 4 month primary end point (23) and another sham control trial targeting the subcallosal cingulate (SCC) was terminated due to lack of significant antidepressant efficacy at 6 month clinical endpoint in a futility analysis (13). The failure of sham controlled DBS trials can be attributed to several factors, such as micro-lesion/insertional effects in the sham arm, placebo responses related to invasive procedures, inadequate study duration, poor patient selection, imprecise targeting or inadequate knowledge of ideal electrode locations, and unknown optimal stimulus parameters.

It is well recognized that adjustments in stimulation parameters and precise targeting are pertinent for optimizing DBS clinical outcomes (5, 7, 11, 31). The determination of the optimal stimulation parameters is crucial: (1) to improve clinical efficacy; (2) to minimize side effects; (3) to maximize the battery life; and (4) to evaluate the dose-response relationship between stimulation parameters and clinical effects (32). At present, the selection or determination of optimal stimulation parameters for DBS in depression is not empirically based, but adapted from movement disorders. Yet, the targets of DBS for movement disorders are cellular nuclei, whereas some of the targets for TRD are white matter pathways such as SCC and medial forebrain bundle (MFB). Typical DBS parameter settings for movement disorders range from 2 to 4 V amplitude, 60–450 us pulse width, 130–185 Hz frequency (32). Optimal stimulation parameters for movement disorders may vary depending on target site, such as sub-thalamic nucleus or globus pallidus, and conditions, such as Parkinson's disease, tremor, or dystonia (32). However, for movement disorder DBS there is often an immediate or early obvious response to observe, such that electrical dose titration is well established (33, 34). Given the lack of empirical data on stimulus response relationship for TRD, a knowledge gap about the optimal stimulation parameters for future studies exists.

The goals of this review are: (1) to map the existing literature and identify the current state of evidence on stimulation parameters of DBS for TRD, (2) to provide a clinical perspective on their relevance to efficacy, safety, and brain targets, and (3) to identify research gaps and present suggestions for future studies to determine if optimal stimulation or electrical dose adjustment is crucial for clinical response.

## Background

### DBS programming and optimization

Stimulus optimization is designed to maximize the clinical outcomes and minimize side effects and is achieved by altering the stimulation parameters of frequency, pulse width, amplitude (voltage or current), and polarity (mono-, bi-, or tri-polar). The electrode contacts can also be changed for precise targeting to maximize outcome with minimal stimulation. A basic understanding of the biophysics of these stimulation parameters on neural tissue is necessary for any clinician who is involved in manipulating the settings and multiple papers have reviewed this topic in the context of movement disorders (32, 34). For this reason, we will only briefly address these issues.

#### Stimulation parameters

The initial papers on selection of DBS parameters used in movement disorders came from the Grenoble team. In their early seminal study, the optimal frequency to suppress tremor with an electrode in thalamus occurred between 150 and 1,000 Hz (35). Limousin et al. (36) demonstrated that to reduce akinesia and rigidity in Parkinson's disease the frequency of DBS in the subthalamic nucleus needed to be above 50 Hz and was maximal around 130 Hz. There was a further non-linear relationship between clinical efficacy and frequency up to 185 Hz. The selection of 130 Hz as a standard frequency used in Parkinson's disease (PD) was a compromise between power consumption and clinical efficiency.

Pulse width is often selected based on biophysical principles. A shorter therapeutic pulse duration (60–90 us) is commonly selected for DBS because it can increase the therapeutic window of voltage changes (37), minimize charge, thereby decreasing power consumption, and increase the threshold difference between activation of different diameter nerve fibers (38) as well as those at different distances from the electrode (39).

After selection of frequency and pulse width, amplitude (either voltage or current) is titrated to achieve the best balance between clinical efficacy and adverse effects. While constant current stimulation systems have become recently available and preferable as it adjusts the voltage as impedance changes (40), much of the existing literature used only constant voltage devices. Whereas, there are slight differences in these 2 systems, the general concept is that the amplitude of both will be increased if insufficient clinical response is obtained. In this article we will use the term “amplitude” to signify either current or voltage.

If amplitude titration does not achieve the best clinical response, pulse width and frequency are manipulated with the same goal in mind. Higher amplitudes and longer pulse width stimulation increases the electrical charge (product of Pulse width × amplitude), charge density (electrical charge divided by the geometric surface of the electrode 0.06 cm2) and activation radius around the electrode contact, by involving more neural elements (axons, dendrites, and cell bodies) (32, 41). This is likely responsible for the observed clinical improvement or side effects associated with progressive current/voltage or pulse width increases with chronic DBS. Current density decreases with distance from the electrode and the responsiveness of neural elements decreases as the distance from the electrode increases (32).

#### Stimulation mode

Two stimulation modes are commonly applied: monopolar and bipolar. In each configuration, current flows from the anode to the cathode, depolarizing the neural elements close to cathode and hyperpolarizing the neural elements close to anode. Cathode is a negative electric potential (sink of current) and an anode is positive electric potential (source of current). In the monopolar setting, the implanted pulse generator acts as the anode and one or more electrode contacts are programmed as cathode. For bipolar stimulation, one electrode contact is the anode and another contact is set as cathode. The monopolar stimulation produces a higher volume of tissue activated around the cathodal pole in a roughly spherical volume, compared to bipolar stimulation, which is more ellipsoid around the cathodal contact. Monopolar stimulation is usually tested first as it requires lower intensities to achieve therapeutic efficacy. However, it is also associated with greater side effects due to wider spread of the current. Therefore, if stimulation related side effects occur, switching from monopolar to bipolar may be a solution if a reduction of monopolar amplitude fails to resolve the side effects.

## Methods

**Figure 1:**
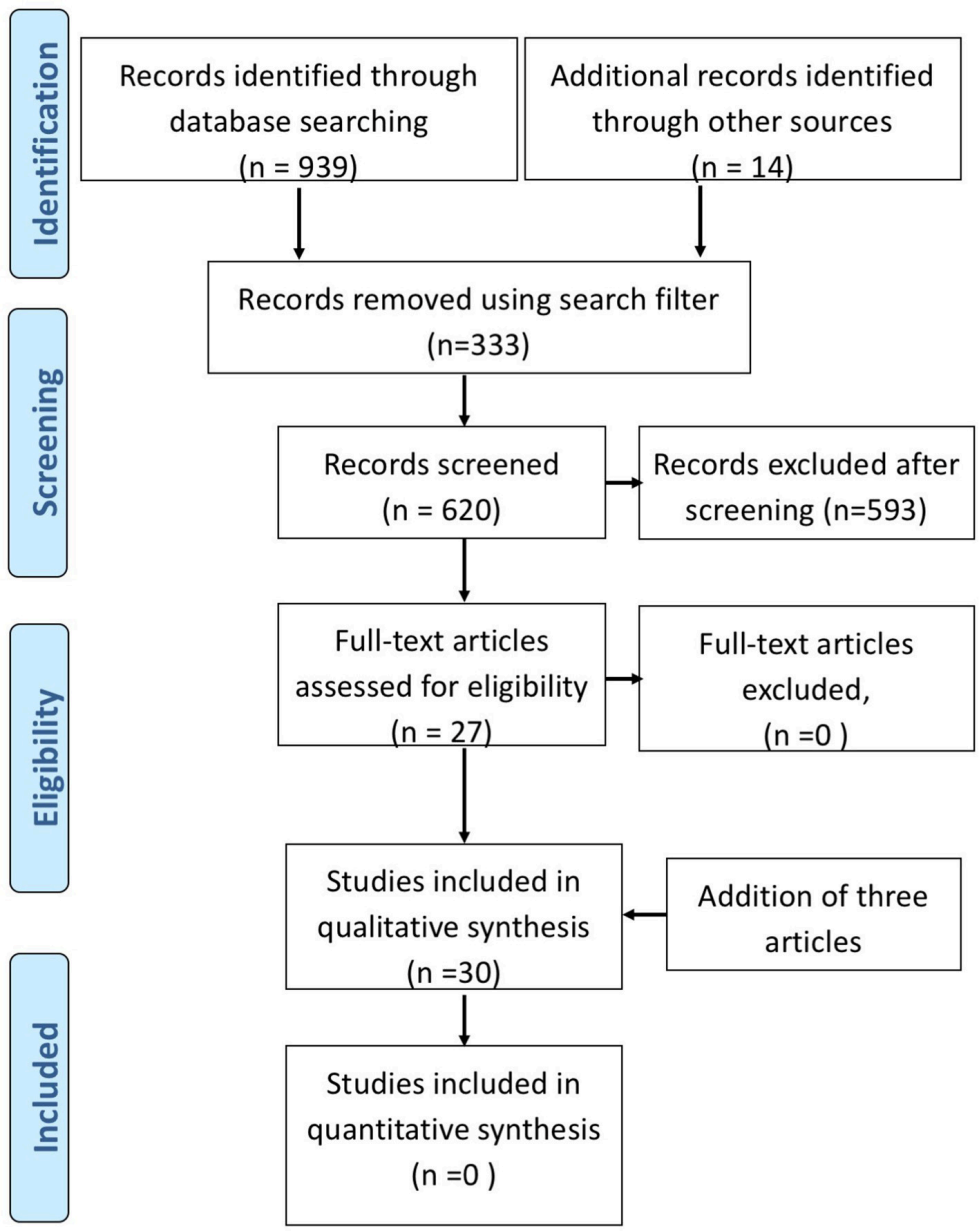
PRISMA flow diagram to identify relevant literature.

Literature search was performed on published data archived in 4 databases: Ovid Medline (1946-), Ovid Embase (1980-), Ovid Psych INFO (1806-), and Scopus (1823-) until July 2017. The search was performed on July 25, 2017. Our search strategies combined subject headings and text words for concepts related to DBS and TRD. The search words used were; TRD, major depression, bipolar depression, DBS, brain depth stimulation, stimulation parameters, brain electrode implant. We applied search filters to exclude animal studies, comments, editorials, letters and review articles, and limited results to English language records. Our search retrieved a total of 939 records from electronic databases, and 11 items from reviewing reference lists of relevant publications. We removed 322 duplicate records, and screened the titles and abstracts of 617 unique records. From 617 abstracts, 593 were excluded. The excluded abstracts consisted of animal studies, review articles, articles on other types of interventions (cortical stimulation), and non-invasive stimulation (transcranial magnetic stimulation, vagal nerve stimulation, electro-convulsive treatment), and articles not focused on depression. Clinical trials (open label and sham controlled trials), case series, and case reports referring to DBS treatment in depression with descriptions of stimulation parameters and clinical outcome were included. We included articles on primary TRD and depression co-morbid with OCD and movement disorders. Any repetition of reports involving original cases describing cognitive or neuropsychological outcomes were identified and removed. The articles using the same patient cohorts reporting on short and long term outcomes separately as individual papers were included because electrical parameters may have changed over time. Furthermore, articles reporting outcome of additional new patients along with previously published data of original cohorts, were included. The articles on physics of electrical stimulation, computer models, were not reviewed and only presented in context to clinical aspects of stimulation. In all, we identified 27 full text articles on DBS for both TRD and co-morbid or secondary depression (see PRISMA Figure 1). After the completion of the literature search, an additional 3 DBS studies on TRD were published during the preparation of the manuscript (13, 14, 42). These were included in the final manuscript. We extracted specific data pertinent to stimulation parameters, mode of stimulation, stimulation adjustments, electrode contacts, location, methodological design (sham control, double blind on-off clinical efficacy), and adverse effects related to stimulation.

## Results of stimulation dosimetry of DBS studies for TRD

**Figure 2:**
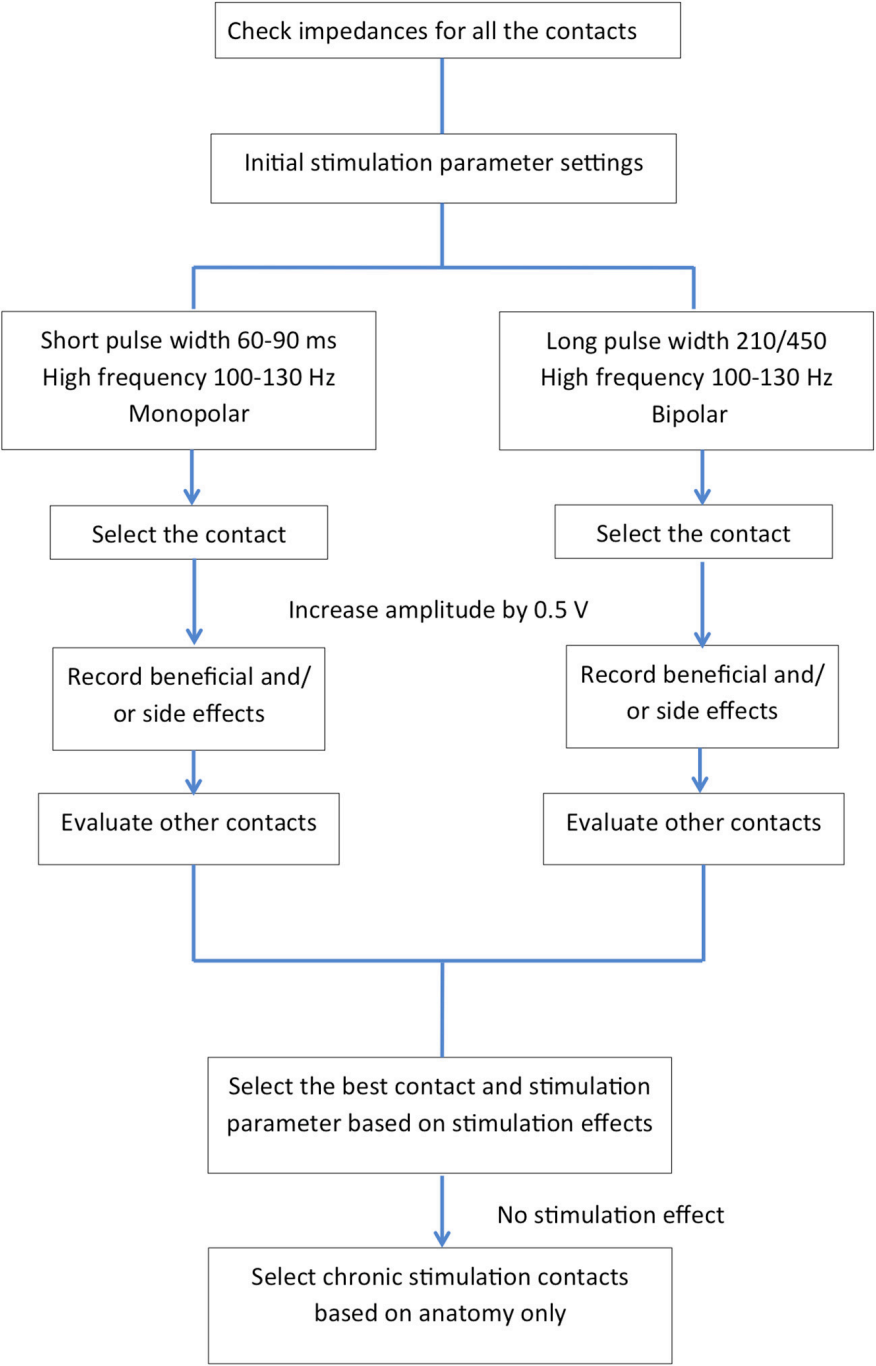
Common algorithms used in the published literature for initial programming (intra-and post-operative programming).

**Figure 3:**
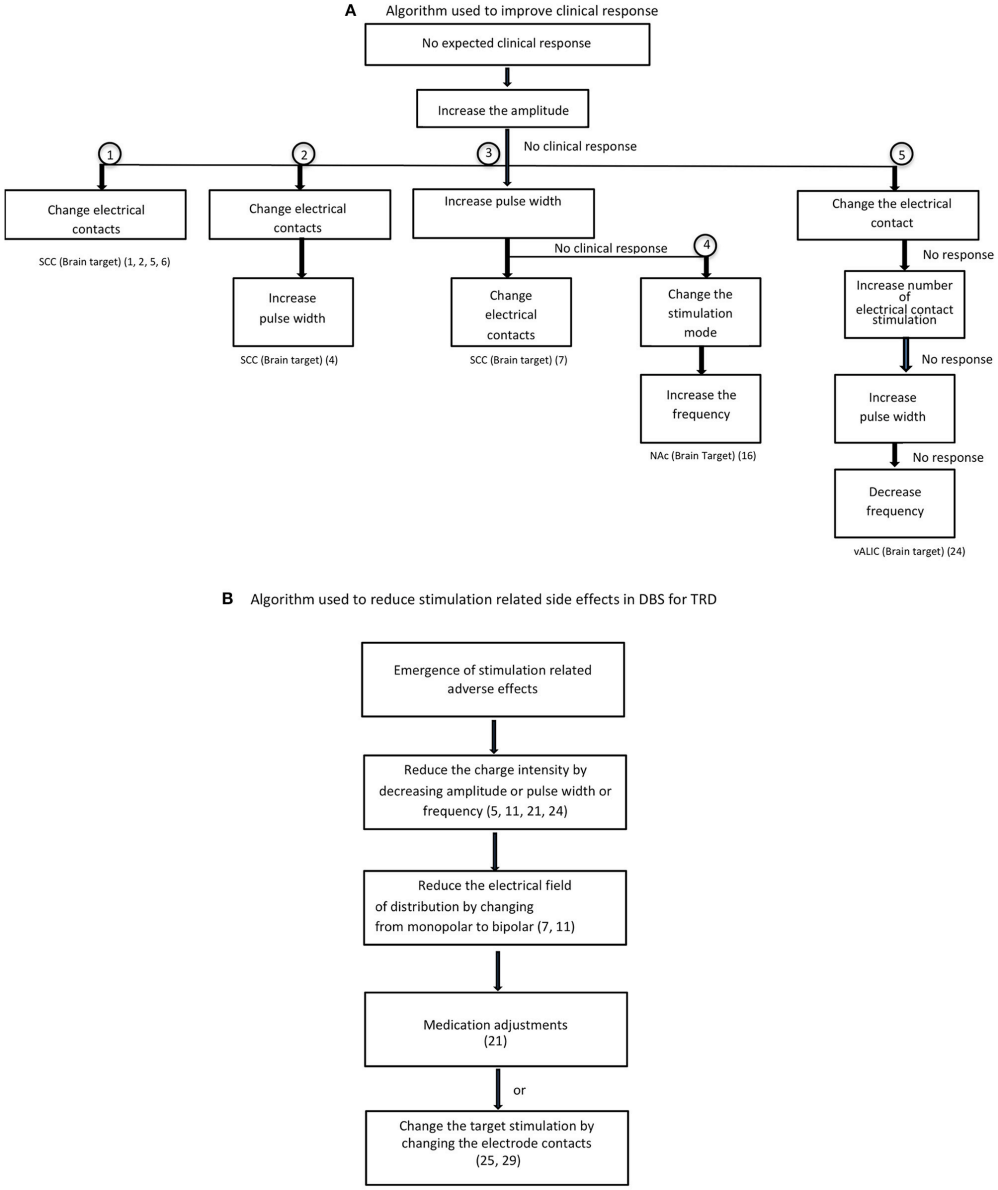
Optimization of stimulation. **(A)** Algorithm used to improve clinical response. **(B)** Algorithm used to reduce stimulation related side effects in DBS for TRD.

Table 1 summarizes the published literature on DBS studies for TRD including the DBS brain targets, stimulation parameters, stimulation mode used and clinical outcomes. Figures 2, 3 depict algorithms of stimulation used for initial programming and during chronic stimulation.

**Table 1:** Stimulation parameters of DBS studies on TRD.

**References**	**Number of patients and diagnosis**	**Follow up in months**	**Stimulation mode**	**Stimulation parameters**						**Response/Remission rates**	**Adverse events**
				**Initial setting**			**Optimal setting**				
				**Amp. v**	**Freq. Hz**	**Pulse width μs**	**Amp. v**	**Freq. Hz**	**Pulse width μs**		
**SCC**											
(1)	6-MDD	6	Mono Polar	2–3	130	60	4	130	60	Res-66%	None
(2)	20-MDD [6 patients from Mayberg et al. (1) study]	12	Mono Polar	3–5	130	90	NA	NA	NA	*6-months* Res-60% Rem-35% *12 months* Res-55% Rem-35%	Worsening of mood/irritability-10%
(6)	17-MDD [All patients from Lozano et al. (2) study]	36	Mono Polar	3.5	130	90	4.3	124.7	70.6	*12 months* Res-55% Rem-20% *24 months* Res-45% Rem-20% *36 months* Res-60 % Rem-40%	Completed Suicide-10% Increase suicidal ideation-15% Worsening depression-15% None of the above side effects were considered on stimulation related
(4)	21 MDD	12	Mono Polar	2.5–5	130–140	91	*6 M* 3–7 *12 M* 2.5–7	130 110–130	91–182 65–117	*6-months* Res-48% *12-months* Res-29%	Nusea,voimiting-45% Agitation-15% Completed suicide-5% Attempted suicide-5% Insomnia-5% Headache- 30% Tremor/spasms-20% Dizziness, Polyuria, weight gain-5-15%
(5)	10-MDD 7-BP	24	Monopolar	4	130	91	6–10	130	91	*6-months* Res-41% Rem-18% *12 months* Res-36% Rem-36% *24 months* Res-34% Rem-58%	Worsening dep-11% Anxiety-11% Suicidal attempt-11%- related to psycho social stress Nausea-21% Headache-16% Gait problems, arm weakness, tingling-5-11%
(7)	8-MDD	12	Monopolar at initial setting. Bipolar- optimal setting	3.5	135	90	4.2	135	120–210	*6-months* Res-87.5% Rem37.5% *12 months* Res-62.5% Res-50%	Suicide attempt-12.5% Recurrence of Depression-25% In the first 6 months
(8)	1-MDD	30	Monopolar	4.5	130	60	4.5	130	60	Res-100% At 30 m	None
(9)	6-MDD	6–9	Monopolar	2.5–10	130	90	5	130	90	33% at 6 and 9 months	None
(10)	5-MDD [3-MDD from Merkl et al. (9) study]	6	Monopolar	2.5–10	130	90	5	130	90	No response in the new 2 MDD patients	None
(11)	4 MDD	9	Monopolar	0–10.5	135	60	2–5	130	90–450	Res-50%	Worsening anxiety-50% Insomnia-25%
(12)	1MDD	12	Monopolar	1.5	90	70	4.5 Right	130 Sided	90 Stim-lation	Remitted	Worsening of depression with left sided stimulation
(13) (BROADEN study)	90 MDD Sham 60-Active 30 Sham	Sham-6 Open-24	Monopolar	4	130	91	8	130	91	*6 months* Active-Res-20% Sham-Res-17% *12 months*-Res-29% *18 months*-Res-53% *24 months*-Res-49%	No stimulation related adverse effects Completed suicide 15% in Sham group
(14)	9 MDD	RCT-6 Cross over-6	Monopolar	4 4 4 4	130 vs. 20 130 vs. 20	91 91 91 91				*6 months* Δ HDRS -15.4 ±21.1 Δ HDRS-14.7 ±21.1 *Cross over-6 M* Δ HDRS-31.3 ± 19.3 Δ HDRS -7.7 ± 10.9	No difference in adverse effects between high and low frequency
**NAC**											
(15)	3MDD	6	Monopolar	4	145	90	4	145	90	Res-0 Percent change in all-20-30%	None
(16)	10 MDD	12	Monopolar	1.5	130	90	1.5–10	100–150	60–210	Res-50%	tension/restlessness/erythem a-30-40%, Hypomania-20% Agitation, paresthesia-20% headache, vision& ocular symptoms, psychosis, muscle cramps, dysphagia-10%
(17)	11MDD	48	Monopolar at the initial setting. Then switched to all unipolar bipolar combination during optimization	1.5	130	90	6.8 7.1	130 135.5	90 (Res) 100 (Non -Res)	Res-45%	tension/restlessness/erythem a-40%, Agitation, disequilibrium-30% Mood elevation, paresthesia- 20% Psychotic symptoms, muscle cramps, vision and eye movement disorder, head ache-10%
(18)	1 OCD and MDD	15	Monopolar	2	130	90	4	130	120	Dep-RemittedOCD-improved	None
(19)	2 OCD& MDD	15	Monopolar	2	130	90	4	130	120	Dep/OCD remitted with both NAcc and Ventral caudate stimulation	None
(20)	4 MDD	9	Monopolar	4	130	60	5–8	130	60	Res-75%	Attempted suicide-25% Increased appetite, food intake, libido- 25%, Worsening of mood and anxiety-50%
**VC/VS**											
(21)	14–MDD 1–BPD	6–51	Monopolar	NA	100 or 130	90 or 210	6.7	127	113	*6 months* Res-40% Rem-20% *Last follow up* (8–51M) Res-53.3% Rem-40%	Hypomania-13.3% Increased depression/suicidal behavior-13.3% Hypomania-improved with stimulation adjustments
(22)	2–MDD patients added to the original cohort of 15 MDD	14–67	Monopolar	2.5–8 V	100–130	NA	NA	NA	NA	*3 months* Res-53% Rem-35% *6 months* Res-47% Rem-29% *12months* Res-53% Rem-41% *Last follow* Res-71% Rem-35%	Paresthesia, anxiety, mood changes, autonomic effects Reversed with stimulation Changes
(23)	30–MDD	Sham 4 Open label 24	Bipolar Monopolar & Bipolar	0–8 NA	NA NA	90 or 210 NA	NA NA	NA NA	NA NA	*4 months* Active stimulation Res-20% Sham 14% *12 months* Res-20% *18 months* Res-26.7% *24 months* Res-23.3%	During active stimulation: Mania/Hypomania-267% Suicide attempt, Disinhibition-13%,Suicide attempt Suicidal ideation-13% Suicide 3 % (Not related to stimulation) Worsening depression-26% Suicide attempt-13% Suicidal ideation-16%
**ALIC**											
(24)	25-MDD	Optimi zation 12 blinded cross over 3	Monopolar	3.5	180	90	6	180	90	Res-40%	Transient mania/hypomania- 12%, Excessive talking- 24%, Flight of ideas-4%, Increased libido-4%, Agitation-28%, Restlessness-24%, Headache 20%, Increased sweating- 12%, Sleep disturbances- 8%, Completed suicide-8% in non-responders, Suicide attempts-16%
**ALIC AND ITP**											
(42)	7 MDD	Double Blind Crossover Between two targets 36 and 96	Monopolar	1–8	130	90/210	3–9	100–130	60–330	Significant decrease in depressive symptoms	2 patients suicided 7 patients worsening depression at some point 6 patients sleep disturbances
**slMFB**											
(25)	7-MDD	3–8	Bipolar	2–3	130		2–3.5 4–5	130 130	60 R 60 (NR)	60	Strabismus/blurred vision- 100% at higher amplitudes Dizziness & increased sweating
(26)	4-MDD	1-single blind sham lead in study	Monopolar	2–3	125	75	3	130	60	Res-75%	Ocular side effects with higher Voltage
**ITP**											
(27)	1MDD & Borderline personality disorder	8	bipolar	2.5	130	450	2.5	130	450	Remitted	None
**HABENULA**											
(28)	1MDD	15	monopolar	5	130	60	10.5	165	60	Rem at 15 m	None
**STN**											
(29)	27 Parkinson & Depression	18	Monopolar	1.4	130	60	1.4–3.7	130–185	60–90	Depression improved at 18 m	Increase in voltage worsens the depression
**GPI**											
(30)	1MDD with Tardive dyskinesia	18	Monopolar	NA	NA	NA	3.5 Left 3.8 Right	130	90	Responded	None

### Subcallosal cingulate gyrus target

Three open label studies, three case series, and three case reports have been published. One large randomized controlled trial on SCC-DBS was terminated following futility analysis and results of this trial have just been published (13). Mayberg et al. (2, 6) first reported DBS of the SCC for TRD in six patients (1) and this cohort was subsequently expanded to 20 patients. The detail description of stimulation settings for intra operative programing and chronic stimulation was given in the 6 months follow up study by Lozano et al. (2) Initial intra-operative DBS was applied using monopolar stimulation with a pulse width of 60 μs and a frequency of 130 Hz. Voltage was increased by increments of 1.0 V every 30 s with a 15–20 s pause between adjustments, up to a maximum voltage of 9.0 V. Post-operatively, the patients were discharged with stimulation off. Chronic DBS was initiated 1 week later using the lowest voltage and specific electrode contacts that produced acute behavioral effects. For the following 4 weeks, stimulation parameters were re-assessed weekly with minor adjustments if the patient showed < 10% reduction in Hamilton Depression Rating Scale (HDRS-17) or developed adverse effects. *Adjustments included changing the voltage or the activated contacts*. By final follow-up, stimulation parameter data were available for 17 of 20 patients with 15 receiving bilateral monopolar stimulation and 2 receiving unilateral stimulation. Mean voltage employed was 4.3 V (range 2.5–8.0 V, σ = 1.7), while the average pulse width was 70.6 μs (σ = 14.8, range not reported), and the average frequency was 124.7 Hz (σ = 21.8, range not reported). There were no significant differences between responders and non-responders with respect to stimulation parameters. The authors reported a response rate of 55% and a remission rate of 35% in the intention to treat analysis with a follow-up of 3–6 years.

In a subsequent multicenter trial, by Lozano et al. (4) (*N* = 21), using a constant current pulse generator (St. Jude Medical) the initial selection of stimulation parameters was based on the individual patient responses over a period of 1–2 weeks. At the first follow-up visit, the average current was 4.2 mA (2.5–5.0 mA), pulse width of 91 μs (range not reported), and frequency of 130.5 Hz (130–140 Hz) with the mean number of active contacts being 1.3 on the right (range 1–4) and 1.2 on the left (range 1–3). *Adjustments included changing the voltage or pulse width*. These values changed slightly at the 6 month follow up with a slight increase in average current (4.9 mA, range 3.0–7.0), and pulse width (100.5 μs, range 91–182). At 12 month follow up, the average current was 5.2 mA (2.5–7.0), pulse width was 93.9 μs (65–117), and frequency was 128.1 Hz (110–130). The average number of active contacts on the right was 1.5 (range 1–4) and 1.4 (range 1–4) on the left. Using a reduction in the HRSD-17 of 50% or more as the main criterion, the authors showed an efficacy rate of 48 and 29% of SCC DBS at 6 and 12 months, respectively.

In the study by Holtzheimer et al. (5) (*N* = 17) the initial stimulation parameters for chronic DBS were monopolar configuration with a frequency of 130 Hz, a pulse width of 91 μs, and 4 mA current. Stimulation intensity was increased to a maximum of 8 mA if there was no clinical improvement. If there was still no improvement after 4 weeks of 8 mA stimulation, the stimulation contact was changed. Pulse width and frequency remained unchanged during the 24-week open-label stimulation phase. After 1 year, the remission and response rates were both 36% which improved to 58 and 92%, respectively after 2 years.

In the study by Puigdemont et al. (7) (*N* = 8) the stimulation parameters were set in the first 1–5 days postoperatively. The initial stimulation parameters were monopolar stimulation at 3.6 V, 135 Hz, and 90 μs. Later they were adjusted based on clinical changes. *The change sequence to maximize therapeutic effect involved sequentially changing voltage, pulse width, and active contacts*. It was determined that bipolar stimulation provided better clinical effects with less side effects, and this configuration was used in the subsequent 5 patients. At 1 year, the response and remission rates were 62.5 and 50%, respectively. All patients were stimulated at 135 Hz. Pulse durations ranged from 120 to 210 μs for responders (with 2 patients using 210, and 3 patients using 180 μs) while amplitudes ranged from 3.5 to 4.5 V. In the 2 non-responders stimulation parameters were 4.5 V, 180 μs and 3.5V, 135 μs. While such few patients could not demonstrate a significant difference in parameters between responders and non-responders, it seemed that those utilizing longer pulse widths had more benefit. Of note, there was a significant relationship between electrode location and clinical response, with responders having electrodes placed mostly in Brodmann area 24, the corpus callosum, and the head of the caudate while non-responders had electrodes closer to Brodmann area 25.

Ramasubbu et al. (11). investigated the relationship between stimulation parameters and clinical effects in 4 patients with TRD. This study consisted of double blind randomized stimulus optimization phase for the first 3 months and post optimization open label phase for the following 6 months. One week post-surgery, each patient underwent testing for the acute effects of stimulation of each electrode. Stimulation threshold for positive effects on mood and for side-effects were noted by progressively increasing the amplitude while holding frequency and pulse width constant (130 Hz and 60 μs). The electrode requiring the lowest amplitude for eliciting a positive effect, and the highest threshold for eliciting a negative effect was chosen as the active contact. If there were no acute effects of stimulation, then the contact activated was based on anatomic location. During weeks 2–7, different frequencies (0, 5, 20, 50, 130, 185 Hz) were changed weekly in a double blind randomized manner, holding pulse width (90 μs) and amplitude (5 V) constant and assessing clinical mood responses. During weeks 8–11, pulse width (0, 90, 150, 270, 450 μs) was assessed in a similar double blind randomized manner maintaining frequency constant at 130 Hz. To limit the charge density to 30 uC/cm, voltage was decreased to 3 V when pulse width exceeded 150 μs. After the stimulation parameter optimization phase, the parameters that were associated with a 50% reduction in the HDRS-17 and maximal mood response were chosen for the post-optimization phase. These parameters remained unchanged for the following 6 months, apart from a decrease in pulse width or amplitude if negative side effects occurred. During the first 6 weeks, two patients did not respond to different frequencies, one patient experienced maximal increase in positive affect at 50 Hz without a change in depression scores, and another patient showed a 50% reduction in HDRS-17 scores along with a maximal increase in positive affect and decrease in negative affect at frequencies of 20 and 130 Hz. During the pulse width optimization period, all four patients showed a maximal response in mood and three patients experienced a 50% reduction in HDRS-17 scores at these longer pulse widths (270, 450 μs). The optimal stimulation parameters determined by the 12-week optimization phase for three of the four patients were: 2 V, 270 μs, 130 Hz; 2 V, 450 μs, 130 Hz; and 2 V, 450 μs, 130 Hz. Each patient received monopolar stimulation. The fourth patient was a non-responder using all stimulation parameters. Limitations of this study included carry-forward cumulative effect of stimulation over time and a fixed order of frequency changes followed by pulse width changes during this optimization.

In the study by Merkl et al. (9) (*N* = 6), monopolar configuration was used and chronic stimulation was set at 130 Hz, 90 μs, and 5 V. There were no adjustments made during the 9 months of follow up. The best electrode contacts in each patient was determined by a sham controlled 24-h stimulation efficacy evaluation of ventral and dorsal homologous pairs. The response rate at 6 and 9 months was 33% (2 out of 6). None of the 6 patients developed stimulation related side effects even at the maximum stimulation intensity of 10 V during the acute stimulation programming phase. Following this study, Accolla et al. (10) investigated 2 more TRD patients along with 3 patients from the previous study, using the same DBS and stimulation protocol for 24 months. The 2 new patients did not respond to DBS during 2 years follow up.

The BROADEN study sponsored by Abbott (previously St. Jude Medical) was a multicenter randomized sham controlled double blind study of SCC DBS (13). Ninety patients with TRD were randomly assigned to 6 months of active (*n* = 60) and sham (*n* = 30) stimulation followed by 6 months of open label phase. Stimulation was initiated in the stimulation group with the stimulation parameters that included monopolar stimulation at 130 Hz, 91 μs, and 4 mA. Control group received sham programming but no actual stimulation was given. Regarding stimulation adjustment, 2 weeks after the stimulation began, amplitude was increased to 6 mA if the MADRS (primary outcome measure) was < 10% lower than that on the previous evaluation. Then after 4 weeks, the amplitude was increased to 8 mA if MADRS was again < 10% lower than the previous score. After 4 weeks, if MADRS score was < 10% lower than the previous score, the second contact from the preselected contact was added. No modifications were allowed in pulse width or frequency. No changes in the stimulation parameters were made after 10 weeks for the rest of the randomized control phase and following 6 months of open label phase. Only after 12 months were changes in stimulation parameters allowed. Changes in medications and addition of psychotherapy were allowed after the first 6 months. Active stimulation failed to show significant efficacy over sham as the response rates between the two groups were similar (active stimulation-20%; sham-17%). With long term open label treatment, the response rate gradually improved over 24 months (29% at 12 months; 53% at 18 months; 49% at 24 months). In a *post-hoc* analysis, the authors found that stimulation parameter changes increased the proportion of responders (*p* = 0.013) in the 18–30 month open label phase. There were no stimulation related side effects reported. Two deaths occurred in sham group in the first 6 months.

The first multicenter double blind randomized cross-over study investigating the effects of high vs. low frequency stimulation of Cg25 was recently published (14). The authors randomized 9 patients with TRD to receive either high (130 Hz) or low frequency (20 Hz) stimulation in the first 6 months and then the non-responders were crossed over to the other group in the next 6 months. Pulse width (91 μs) and amplitude (4 mA) were fixed for both high and low frequency groups and stimulation setting was monopolar during the trial period of 12 months. After 6 months of active stimulation, the mean percent change in MADRS score showed improvement in both low and high frequency groups but there was no significant difference between groups. After the cross-over period at 12 months, the low frequency stimulation crossed over to high frequency was more effective than the high to low frequency stimulation. There were no differences in the side effects between groups. This study was limited by a small sample size and high attrition rate in the cross-over phase. Among the total of 9 patients only 6 patients participated in the cross-over phase and only 5 were actually crossed over.

In a single TRD patient with a prior cingulotomy, Neimat et al. (8) used bilateral monopolar stimulation at 4.5 V, 130 Hz, and 60 μs and achieved a good outcome with a 68% improvement in HDRS-17. The authors reported minor stimulation adjustments but likely ended up with the same stimulation parameters they started with at the beginning of the study. Guinjoan et al. (12) described successful remission of a TRD patient with SCC-DBS. Initial parameter settings were 1.5 V, 70 μs, 90 Hz with titration upwards to maximum of 6 V, 90 μs, 130 Hz during intra- and post-operative programming. Changes in contacts and stimulation parameters were made during the chronic stimulation period. Bilateral stimulation using parameters set at 120 Hz, 90 μs, and 4.5 V failed to show improvement, whereas stimulation of left SCC worsened the depression and right sided SCC stimulation produced remission.

### Nucleus accumbens (NAc)

NAc is an important target in reward pathway for DBS treatment in TRD. The other target regions of this pathway used in DBS studies including ventral striatum and ventral capsule (VS/VC) and anterior limb of internal capsule (ALIC) are anatomically overlapped with NAc and could be considered as the same brain target. However, for clarity, we discussed the study findings under different target regions as reported by the authors.

Aouizerate et al. (18) reported one patient with OCD and depression who received bilateral NAc-DBS. The lower two contacts were in the NAc, while the two upper contacts were located in the ventromedial portion of the caudate nucleus. After 1 month of chronic stimulation of the NAc contacts at 130 Hz, 90 μs, and 2 V, there was no effect on obsessive symptoms, and a very small effect on depressive symptoms. Then the contacts in the ventromedial caudate nucleus were activated at 4 V, 120 μs, 130 Hz. Here the patient achieved remission of depressive symptoms within 3 months, which was maintained 15 months later, along with a decrease in OCD symptoms.

Using the same target, Schlaepfer et al. (15) reported DBS response in three patients with TRD. Initial stimulation parameters were 4 V, 90 μs, and 145 Hz with a monopolar configuration. Stimulation amplitude was manipulated during the study in a double-blind manner, in 1 V steps from 0 to 5 V. At each change in voltage setting, clinical effects were observed through the completion of a variety of depression rating scales. The authors found a negative correlation between the depression severity scores and stimulation intensity.

In another study Bewernick et al. (16, 17), 11 patients with TRD underwent NAc –DBS and were followed for up to four years. Initial stimulation parameters consisted of monopolar stimulation of the ventral two DBS contacts at 130 Hz, 90 μs, and 2 V. After 1 week “off” stimulation, the voltage was progressively increased from 2 to 4 V. These parameters remained constant for 4 weeks to observe the acute and sub-acute effects. Afterwards, changes were made only if the patient experienced adverse effects or failed to experience anti-depressant effects. Parameter changes were made in a sequential fashion, with changes in amplitude (1.5–10 V), pulse width (60–210 μs), configuration (all possible monopolar and bipolar combinations), and frequency (100–150 Hz) in this order. When optimal settings were found, they remained constant for at least 1 month. Average stimulation parameters on final follow-up were almost the same between responders and non-responders (responders 6.8 V, 90 μs, 130 Hz; non-responders 7.1 V, 100 μs, 135.5 Hz). Forty-five percent (5/11) of patients achieved a response within 6 months, and remained stable for the remainder of the follow-up. The authors recommended against changing parameters more frequently, as they noticed a time lag of 2–4 weeks between stimulation parameter changes and observable clinical effects.

Millet et al. (20) examined the efficacy and safety of DBS involving the NAc/caudate target in 4 TRD patients. Stimulation parameters were fixed at 130 Hz, 60 μs, and 4 V for 8 months. In the extended 6 month follow-up phase, the amplitude was increased to 8 V keeping the same frequency and pulse width. Three of four patients responded at 12 months during the extended period only when voltage was increased. Clinical outcomes of NAc target stimulation were better than the caudate target. One patient attempted suicide, two had increasing anxiety/depression, and one patient had increased appetite, libido, and worsening sleep. It was not clear whether stimulation was adjusted to mitigate these possible adverse effects.

### Ventral striatum/ventral capsule (VS/VC)

In an open label study, Malone et al. (21, 22) examined the effect of DBS involving the ventral striatum/ventral capsule in 15 patients with TRD and followed them for a period of 6 months to 4 years. Intraoperative test stimulation was employed to identify contacts that produced acute improvements in mood without adverse effects. After a recovery period of 2–4 weeks, patients underwent stimulation parameter titration for several days. Each contact was assessed in a single blinded manner to determine effects at each electrode, initially with a monopolar configuration and followed by assessment of select bipolar configurations. Chronic stimulation parameters were based on this initial testing phase. Mean stimulation parameters at last follow-up were: amplitude 6.7 V (±1.8 V), pulse width 113 (±45) μs, and frequency of 127 (±11) Hz. Overall, mean amplitudes tended to be lower in responders vs. non-responders, though this was not statistically significant. To lengthen battery longevity, frequency was lowered in some patients without subsequent change in clinical response. The authors noted that individual assessment of pulse width was important, as changes to this parameter influenced patient response. Symptom improvement was seen with an increase or decrease in pulse width, depending on the patient and the contacts used. Most patients had the distal electrodes programmed as the cathode, in either a monopolar configuration or a bipolar configuration with the most dorsal contact 3 as the anode. At final follow up, 8 patients (53%) responded of which 5 patients (33%) met the criteria for remission.

In a multicenter randomized sham controlled trial (RECLAIM study), 30 patients with TRD received active or sham VC/VS stimulation (1:1) in a double blind fashion for 16 weeks followed by a 24 month open-label phase of active DBS (23). During the blinded phase, high frequency (range was not mentioned) bilateral stimulation with two different fixed pulse widths (90 and 210 μs) and voltage up to 8.0 V was used to optimize the clinical outcome. Bipolar stimulation was used during the blinded phase to reduce side effects and protect the blinding. In the open label phase, changes in stimulation parameters were allowed; however, the mean/range of stimulation parameters was not provided. The response rate of the active vs. sham treatment (20 vs. 14.3%) was not significant and the response rate during open-label continuation phase was 20–23%. Stimulation related adverse effects during the blinded phase and open phases were reported but the details on stimulation adjustments to minimize the adverse effects were absent. Authors did not respond to the request for stimulation parameters.

### Ventral anterior limb of internal capsule (VC)

Bergfeld et al. (24) investigated the efficacy and safety of ventral capsule (VC)-DBS for TRD in 25 patients who underwent open label stimulus optimization phase for 52 weeks. Patients received monopolar stimulation using the 2 middle DBS contacts starting at 3.5 V while keeping the pulse width and the frequency constant at 90 μs and 180 Hz. The voltage was gradually increased in increments of 0.5 up to 6.0 V if there was no response or partial clinical response. In case of poor clinical response or emergence of stimulation-induced side effects at 6.0 V the electrical contacts were switched to dorsal contacts and the stimulation sequence repeated from 3.5 up to 6.0 V. In case of non-response, the electrical contacts were switched to ventral contacts and the optimization procedure repeated. The other steps that were taken to improve the clinical outcome included: addition of contacts, increasing pulse width, low frequency stimulation (i.e., ≤ 60 Hz) and increasing the voltages above 6.0 V. None of the increases in voltage or pulse width (>120 μs) or low frequency stimulation (≤ 60 Hz) were effective. In case of stimulation-related side effects, decreasing voltage, pulse width, or frequency and turning off one of the contacts was successful. Ten patients (40%) were responders and 15 patients (60%) were non-responders at the end of this prolonged optimization phase. Sixteen patients (9 responders and 7 non-responders) entered a randomized crossover sham vs. active stimulation phase. Active stimulation produced significant reduction in HDRS scores compared to sham stimulation. Three patients experienced stimulation related transient mania or hypomania and 8 patients had behavioral activation or hypomanic symptoms such as excessive talking, flight of ideas, and increased libido, all of which resolved with stimulation adjustments.

### Ventral anterior limb of internal capsule (VC) vs. inferior thalamic peduncle

A comparative study with a double blind cross over design investigating the clinical efficacy of DBS of ITP vs. Anterior limb of internal capsule/ bed nucleus of stria terminalis (IC/BST) in 7 patients with TRD has been recently published (42). The study involved two crossover. In the first crossover phase patients received IC/BST stimulation vs. no stimulation in random order and in the second crossover, patients received IC/BST vs. ITP vs. no stimulation. Patients received monopolar stimulation. For initial programming all patients received monopolar stimulation with 130 Hz, and 210 μs. Voltage was adjusted up to 9 V and clinical effects/ adverse effects were evaluated. The optimal parameters used for IC/BST were: 130 Hz, 60–300 μs, 4.5–9 V and the optimal parameters used for ITP target were: 130 Hz, 210–300 μs, 4–8.2 V. During the first cross over, 4 out of 6 (66.7%) responded to ICT/BS. In the second cross over, 4 out of 5 (80%) responded to IC/BST and 3 out of 5 (60%) responded to ITP. The charge densities were higher for ITP stimulation (42.8 μC/cm2) than for IC/BST stimulation (34.3 μC/cm2). IC/BST stimulation seem to have better clinical effects than ITP stimulation. All patients were stimulated at IC/BST at the last follow up 3 years after DBS implantation. Five out of seven (71.4%) were responders. The optimal parameters at the final follow up were: 100–130 Hz, 60–330 μs, 3.6–9 V. Only one patient received short pulse width (60 μs) and 100 Hz stimulation. Most common stimulation related side effects were worsening depression, sleep disturbance and suicidal thoughts. Two patients who responded committed suicide at 3 and 6 years after the DBS implantation.

### Medial forebrain bundle (MFB)

Schlaepfer et al. (25) targeted the superolateral branch of the MFB in 7 patients with TRD. The target was identified by deterministic diffusion tensor MR imaging (DTI) with a seed region in the area lateral to the ventral tegmental area (VTA). Testing for acute effects of stimulation was performed intra-operatively, with 130 Hz, 60 μs, and 2–3 mA monopolar stimulation. Chronic DBS was initiated 1 week after surgery in a bipolar configuration with a constant voltage (initially 2–3 V, with a target current of 2–2.5 mA based on impedance measures). The amplitude was adjusted to maximize clinical benefit. At last observation (12–33 weeks), mean current for responders (*n* = 6) was 2.9 mA (range 2.4–3.5 mA on left brain, 2.3–3.1 mA on right brain). Among the six responders 4 were remitters. The one non-responder was stimulated at a higher current (4.8 mA). Pulse width and frequency were kept constant at 60 μs and 130 Hz. Fenoy et al. (26) published the results of 4 TRD patients who underwent DBS of MFB following the same procedures as proposed by Schlaepfer et al. (25). Intraoperative testing was performed with 125 Hz, 75 μs, and 2–3 mA. Three of four patients responded at 7 days post-stimulation. Two patients continued to have significant improvement at 26 weeks. One responder withdrew from the study. Stimulation parameters of responders at 7 days were 130 Hz, 60 μs, 3 V. The non-responder had lower stimulation on the left brain (1.9 V) than the right (3.2 V) due to temporary ocular side effects. At 6 months stimulation parameters were 3.2 V, 130 Hz, and 60 μs on both sides.

### Lateral habenula

There is a report of a patient with TRD who underwent DBS targeting the afferent bundle of the lateral habenula (28). The pulse width was fixed at 60 μs, but frequency was increased from 130 to 165 Hz and the voltage was increased from 1.4 to 10.5 V to attain clinical remission.

### Inferior thalamic peduncle (ITP)

Jimenez et al. (27, 43) reported two TRD patients with ITP-DBS. Acute bipolar stimulation was performed increasing the amplitude (1.0–6.0 V), using a fixed frequency of 130 Hz, and pulse width of 450 μs. During this time, the patient was evaluated clinically for adverse and beneficial clinical effects. Chronic bipolar stimulation was performed at 130 Hz, 450 μs, and 2.5 V for one patient and at 3 V for the second patient. One patient achieved remission at month 8, and the other achieved remission at month 18, during a 24 month follow-up period.

### Globus pallidus pars interna (GPi)

Kosel et al. (30) reported the case of a 62 year old patient undergoing bilateral GPi DBS to treat neuroleptic induced tardive dyskinesia and concomitant TRD. Optimal parameters were determined after 6 weeks of testing. The left GPi was stimulated in a monopolar configuration at 3.5 V, 90 us, and 130 Hz and the right contact was stimulated at 3.8 V, 90 us, and 130 Hz. The dyskinesia as well as depression improved substantially at 18 months post DBS. The depression severity as measured by HDRS was reduced by 50% compared to the baseline at the clinical end point. However, it is unclear how much of the improvement in depression was related to improvement in motor symptoms.

### Subthalamic nucleus

The concomitant effects of STN DBS for Parkinson's disease on depression were studied in 27 patients by Wang et al. (29). The range of stimulation parameters was reported, but patient specific stimulation information is unavailable. Stimulation adjustments were likely performed to optimize PD features and therefore are not clearly described. The stimulation applied was: monopolar stimulation with voltages of 1.4–3.4 V, pulse width of 60–90 μs, and frequency of 135–185 Hz. Depression rating scores worsened with increases in mean bilateral voltages. HDRS scores showed a significant decrease in the STN-DBS group at three3 months post-operatively compared to medication control group, though the change was not significant at 6, 12, and 18 months. Scores on the Sheehans disability scale, (a self-reported visual analog scale that measures functional impairment in work/school, social and family life) were significantly reduced in the STN-DBS group at 6 months post-operatively, but no significant effect was seen at 12 and 18 months. It remains unclear whether the antidepressant effect of STN-DBS was dependent or independent of improved Parkinson's disease scores.

## Discussion

**Figure 4:**
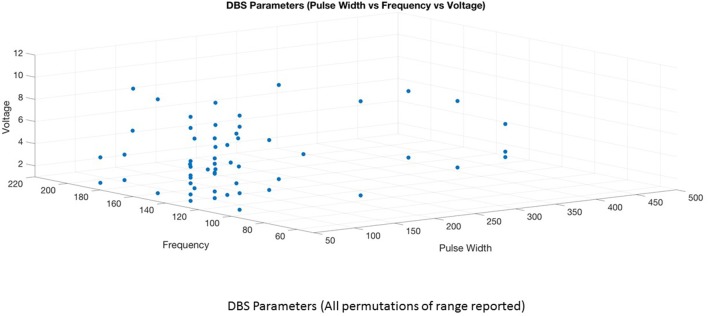
DBS parameters (Pulse width vs. Frequency vs. Voltage).

**Figure 5:**
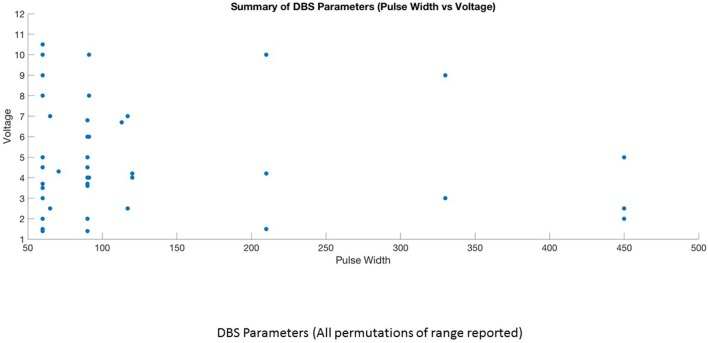
Summary of DBS parameters (Pulse width vs. Voltage).

This is the first systematic review of stimulation parameters used in DBS studies for depressive disorders. Because available evidence on stimulation parameters used in these trials is limited, informed decision-making for rational selection and adjustment of stimulus parameters is difficult. Despite these limitations, we can highlight several important issues. (1) There is heterogeneity in the selection of stimulation parameters and stimulation adjustments during the optimization phase of treatment. The choice of stimulation parameters or adjustments varies depending on the target site, clinician preference and experience. (2) Although it is possible to have an enormous number of different parameter settings (12,964) (32), three optimal stimulation parameter combinations emerged from this review (Figures 4, 5). High frequency stimulation (>100 Hz) was applied in almost all cases. Three different combinations of pulse width and amplitude were commonly used: (i) short pulse width (60–90 μs) with low intensity (2–5 V or mA), (ii) short pulse width (60–90 μs) with high intensity (5–10 V or mA), and (iii) long pulse width (120–450 μs) coupled with low intensity (2–5 V or mA). (3) Clinical response to electrical dosing varies across different levels of charge intensity of electrical stimulation. This review showed the clinical benefit of using both higher charge density stimulation (higher amplitude and longer pulse width stimulation) as well as lower charge density stimulation. This heterogeneity in clinical response to electrical dosing emphasize the need for patient specific approaches in determining optimal stimulation (44). (4) Stimulation related side effects in DBS for TRD were predominantly emotional and behavioral in nature, related to upward titration of pulse width, amplitude (in monopolar stimulation), and specific to brain targets (45).

### Variations in stimulation adjustments

The manual adjustment of stimulation to attain optimal clinical outcome is a more complex and time consuming process for TRD than for movement disorders (46). This is partly due to a lack of consistent acute clinical or behavioral effects of the stimulation in the operating room or during the optimization period (7, 11), a time lag minimum of 2 weeks between stimulus adjustment and clinical response (5), and inter-individual variability in clinical response or in adverse effects to stimulation adjustment. Additionally, the lack of empirical data on the relationship between specific stimulation parameters and clinical or behavioral response, limits the clinicians' ability to select the optimal stimulation parameters at the outset. Furthermore, the average length of time required for optimization in DBS for TRD remains uncertain. In the published DBS studies for TRD, the optimization period varied from 1 to 12 months. In a recent discontinuation study, the optimization period was extended to 12 months (24) whereas the failed double blind randomized control studies set the primary clinical end points at 4 and 6 months, including the optimization period (13, 23). Based on these failed clinical trials, investigators now suggest prolonged optimizing periods between 6 and 12 months (23, 24).

### Variations in stimulation adjustment algorithm

In the stimulation adjustment algorithm (Figure 3A), increases in amplitude have been employed as a first intervention step to improve the clinical response to DBS in most of the studies regardless of brain target. The second step was either to change electrical contacts (5) or increase the pulse width (7, 11, 42). The role of changing electrical contacts to improve outcome was substantiated from two studies reporting an association between electrode contacts and greater improvement (7, 9). A change in electrode contacts may improve the precise targeting of therapeutic fiber tracts within the target region since the electrode contact selection based on initial programming may not be accurate as it relies on clinician preference in the absence of acute behavioral effects during the programming. The third step is to change the mode of stimulation (16) or increase the number of active contacts (from 2 to 8) (24). Increase in frequency as a first step followed by increases in voltage were used for the habenula target (28). Decreasing the frequency to 60 Hz or below was tried, but failed to improve the clinical response in VC-DBS (24). The commonly reported change sequence used to maximize therapeutic effect in SCC-DBS is sequentially changing amplitude, then electric contacts or pulse width (Figure 3). For other DBS targets, there is no similar algorithm used for subsequent steps in optimization in refractory depression, and is predominantly driven by clinician experience.

Psychiatric and physical adverse effects have been reported during stimulus optimization of DBS for TRD (Table 1). The common psychiatric adverse events were mood disturbances such as hypomania or mania, especially in DBS of reward pathways (VC/VS, NAc) (16, 17, 21, 23), worsening depression, anxiety (tension, restlessness, autonomic changes) and insomnia regardless of brain targets. The reasons proposed for stimulation related adverse events include overstimulation and the spread of current beyond the optimal target, or stimulation directed to non-therapeutic fiber tracts. Minimization of stimulation related side effects (Figure 3B) was achieved by reducing the stimulation or spread of the current by decreasing the amplitude, pulse width or frequency (5, 11, 21, 24), changing the mode of stimulation from monopolar to bipolar (7, 11), and changing electrical contacts (25, 29). In some instances, medication adjustment was used to counteract the side effects of mania and hypomania (21). Completed suicides and suicide attempts have also been reported in DBS responders and non-responders and in sham groups not receiving active stimulation (4, 6, 13, 23, 24). It is uncertain it there is a relationship between increase in stimulation and emergence of suicidal behaviors. Since the suicidal rate of 10–20% reported in DBS studies is comparable to suicidal rate in TRD patients, it is possible that the suicidal behaviors associated with DBS treatment may be related to the severity of the base condition (i.e., refractory depression), or DBS treatment failure in non-responders. Furthermore the reported suicide in sham group and in non-responders after the termination of stimulation suggest that suicides in DBS patients are not stimulation related (13, 23). However, the reasons for completed suicides in DBS responders remain an enigma. Future studies should carefully examine the temporal relationship between stimulation setting changes, worsening depression, and emergent suicidal behavior as well as between stimulation and increase in impulsivity and suicidal behavior and the reversal with decrease in stimulation. In clinical practice, suicidal events and worsening of depression or impulsivity during optimization of stimulation need careful monitoring/evaluation and appropriate stimulation adjustments. Among the physical symptoms higher voltage dose dependent ocular side effects were reported in MFB-DBS (25, 26).

### Stimulation parameter combinations

#### High frequency vs. low frequency

High frequency stimulation (100–130 Hz) was used in all parameter combinations regardless of target sites. Additionally even higher frequencies (130–180 Hz) were applied in STN for depression secondary to Parkinson's disease, VC, habenula, and NAc (15, 24, 28, 29). It seems that higher frequency stimulation is used in brain targets with cell bodies (STN, NAc, habenula) except VC which is a white matter target. The only clinical study comparing the effect of low (20 Hz) vs. high (130 Hz) frequency stimulation showed no group differences in clinical efficacy during the first 6 months; however in the following 6 months, the group that was switched from low to high frequency stimulation showed better clinical improvement than those going from high to low frequency stimulation, suggesting that high frequencies are better (14). Preclinical studies in rodents has also been in favor of high frequency stimulation: only high frequency stimulation (130 Hz) of the ventromedial prefrontal cortex (analogous to SCC in humans) yielded optimal antidepressant effects compared to low frequency stimulation (50 Hz) (47). Overall the available evidence suggests that higher frequency stimulation at 130 Hz yields better outcomes than lower frequency stimulation.

#### Combinations of pulse width and amplitude

Keeping the high frequency constant, 15 studies utilized short pulse width—low intensity (1, 2, 4, 6, 8–10, 12, 14, 15, 20, 25, 26, 29, 30) 6 studies employed short pulse width—high intensity stimulation (5, 13, 17, 20, 24, 28), while 7 studies used long pulse width—low intensity stimulation (7, 11, 16, 18, 19, 21, 42). Three studies used long pulse width—high intensity stimulation in few patients (16, 21, 42) and two studies could not be classified due to lack of information on optimal stimulation parameters (22, 23). The average response rate at 6–9 months for short pulse width—low intensity was 53% (range 33–85%) and at 12 month was 45% (range 28–55%). Two studies involving 2–3 cases reported zero response rate at 6–9 months (10, 15) and three case reports of long term outcome showed 100% response rate (8, 12, 30). In the short pulse width—high intensity group, the average response rate at 6–9 months was 31% (range 20–75%) and longer term (>24 months) was 54% (range 45–92%). The average response rate at 6 months for short pulse width—high intensity stimulation was only 20% in the sham controlled BROADEN study. The average response rate for long pulse width—low intensity group at 6 months was 81% (range 50–100%). There were 3 case reports in this group reporting 100% response (18, 19, 27). The higher response rates in the long pulse width—low intensity stimulation group is likely due to small number of patients. As there are huge variations in response rates within and between groups, it is not possible to compare the groups or conclude the superiority of any particular parameter combination. More importantly, non-stimulation factors may contribute to the inconsistencies in response rates. For example, in the short pulse width—low intensity combination, studies targeting MFB showed better response rate at 6 months (75–85%) than SCC-DBS studies (33–66%). In short pulse width—high intensity group, the sham controlled trial (BROADEN study) involving larger sample size produced an overall poor response rates compared to open trials. In the same group, the longer term response rates are better than the 6 months response rates, implicating the role of adjustment in medication, change in stimulation parameters, for clinical improvement. Interestingly, the stimulation parameter change was associated with a significant increase in response rate whereas medication change was not (13).

Currently, the rationale for the selection of 3 different combinations of pulse width and amplitude (short pulse width –low intensity, short pulse width- high intensity and long pulse width—low intensity) is driven by clinician experience, choice and electrophysiological principles. Both high intensity and long pulse width stimulation increases the spatial distribution of the electric field and the volume of tissue activated (41), thereby increasing the recruitment of fiber tracts or neuronal bodies. Given the variations in the anatomy and fiber tracts in the SCC and other targets, increasing the volume of tissue activated either by the amplitude or duration of pulse width should increase the likelihood of clinical improvement. Long pulse width stimulation may have theoretical advantages related to the chronaxie of the neural elements involved (48) while high intensity stimulation may activate both target and non-target neural elements (49). Longer pulse widths may increase the spatial distribution of electric field in non-homogenous regions such as the junction of gray and white matter in targets such as the SCC (50). These theoretical attributes support for a role for long pulse width stimulation in the algorithm of stimulation optimization for DBS-TRD. However, future comparative studies are needed. The combination of long pulse width and high intensity stimulation is less commonly used as this combination increases the charge density above the recommended threshold (charge density 30 μC/cm2) increasing the risk of tissue damage. To keep the stimulation within the charge density limit, previous studies used short pulse width-high intensity and long pulse width –low intensity combinations. The combination of short pulse width-low intensity stimulation is commonly preferred based on the premise that if precise targeting is achieved, lower electrical charge or charge density is sufficient to activate the specific brain region. More importantly this combination is efficient as it decreases power consumption and reduces the risk of side effects.

### Variations in clinical response to electrical dosing

The DBS trials for TRD showed variations in clinical response to electrical dosing as some patients clinically improved with lower current density stimulation (Short pulse width-low intensity stimulation) whereas other patients required higher current density stimulation (High intensity or Long pulse width stimulation) to attain clinical recovery. Given these variations in clinical response, patient specific computational models of stimulation have been proposed to determine or select the optimal stimulation parameters for each individual patient at the early stage of DBS treatment. The other variation in clinical response is related to the time of clinical recovery or the minimum duration of stimulation required for clinical improvement. For example ~50% of patients with SCC-DBS showed response within 6 months (early recovery) while some showed improvement only after 2 years of stimulation (delayed recovery). There is evidence that continuation of low current density stimulation (SPW-LI) or higher density stimulation (SPW-HI) over a longer period (2–4 years) of SCC-DBS stimulation may yield incremental clinical benefits (5, 6, 13). Future comparative studies are needed to address the differential effects of low vs. high current density stimulation on short term and longer term clinical efficacy.

Although upward titration of electrical stimulation was commonly used to optimize the clinical outcome in the previous studies, this review uncovered that most of the studies failed to report on the correlation between electrical dosing and clinical response using quantitative data analysis. To our knowledge, only one study documented a statistically significant negative correlation between increase in amplitude and decrease in HAMD scores (25). Failure to report the relationship could be due to negative results resulting from small sample size or the use of responders and non-responders as one group in the analysis. Our attempt to generate a graph to examine the correlation between charge density and symptom severity was in vain because individual data on changes in stimulation parameters and symptom severity were not reported in major studies. There is a need for quantitative evidence to provide rationale for increasing the current density as a clinical strategy to optimize the clinical outcome. Future studies should examine the relationship between increase in charge density and decrease in symptom severity particularly in DBS-responders.

There are limitations in using higher current densities. Several studies that compared stimulation parameters in responders and non-responders showed no significant difference in parameters (2, 4, 5). In fact one study reported higher intensity stimulation in non-responders, likely because their parameters were pushed up to attempt to achieve response (21). So higher current density stimulation does not always lead to better response in all patients. Of course other factors such as age (21, 23), illness subtype (15), and integrity/ precision of fiber tracts (31) targeted by stimulation may outweigh the influence of electrical parameters in determining response to DBS. The other shortcoming of high intensity or long pulse width stimulation is battery usage resulting in frequent pulse generator replacements. Even if rechargeable systems are used, it can take 1–2 h to charge the battery every day causing inconvenience to the patients. Furthermore, long pulse width and high intensity stimulation may be associated with increased risk for adverse events (7, 21).

### Limitations of reviewed studies

Several limitations common to most of these published studies also limit our interpretation of them. These include a relatively small number of patients, open label design [except four sham controlled studies (13, 23, 24, 41)] and insufficient data reporting about adjustments made in stimulus parameters. Placebo effects will always confound open label trials. Reporting only mean and standard deviation or even range in stimulation parameters is difficult to interpret in the context of individual patients. Some studies failed to report on range of stimulation parameters used to optimize the clinical response (23, 42). With the push to open access data reporting, journals should have authors publish all details especially with new investigational therapies. Only 2 studies reported the criteria defining poor clinical response requiring stimulation adjustments during optimization (5, 13). Furthermore, only a few studies clearly documented the algorithm for stimulation adjustments (1, 5, 7, 13, 24).

## Conclusions

This systematic review has identified the paucity of empirical research in determining the best stimulation combinations, stimulation optimization algorithms, and interaction between stimulation and non-stimulation factors (age, illness subtypes, brain target, integrity of fiber tracts) in DBS response and non-response in TRD patients. Successful DBS requires optimal electrical stimulation of the chosen anatomical target. This review suggests that high frequency is always required, and short pulse width—low intensity, short pulse width—high intensity, as well as long pulse width—low intensity stimulation are all possible combinations for clinical effect. Controlled studies are needed to assess the comparative efficacy and safety of these three combinations, which emerged from this review. A registry for DBS studies for TRD with individual patient data on stimulation parameters and standardized reporting of initial programming and chronic stimulation parameters will advance research in this area. While automated computational modeling of current densities and volumes of tissue activated are now available, new electrodes with directional capabilities, and closed loop DBS systems are on the horizon, these still require a better understanding of which electrical parameters are best for TRD. Prior to embarking large-scale randomized sham controlled trials on DBS for TRD, empirical evidence from clinical studies is needed to determine optimal stimulation parameters.

## Author contributions

RR contributed to the conception of this review, literature search, selection of relevant articles, and wrote the first draft. SL wrote sections of the manuscript. ZK revised the manuscript critically for the intellectual content and the format. All authors contributed to the revisions, read and approved the submitted version.

### Conflict of interest statement

The authors declare that the research was conducted in the absence of any commercial or financial relationships that could be construed as a potential conflict of interest. The reviewer AW and handling Editor declared their shared affiliation.
